# Case report: Unilateral GPi DBS in secondary myoclonus-dystonia syndrome after acute disseminated encephalomyelitis

**DOI:** 10.3389/fneur.2023.1238743

**Published:** 2023-09-26

**Authors:** Alexander Calvano, Laura Beccaria, Lars Timmermann, Miriam H. A. Bopp, Marko Gjorgjevski, Christopher Nimsky, David J. Pedrosa

**Affiliations:** ^1^Department of Neurology, Philipps-University Marburg, Marburg, Germany; ^2^Center for Mind, Brain and Behaviour (CMBB), Marburg, Germany; ^3^Department of Neurosurgery, Philipps-University Marburg, Marburg, Germany

**Keywords:** myoclonus-dystonia, acute disseminated encephalomyelitis, deep brain stimulation, neuromodulation, connectome analysis

## Abstract

**Introduction:**

Deep brain stimulation (DBS) is an established and effective therapy for movement disorders. Here, we present a case of secondary myoclonus-dystonia syndrome following acute disseminated encephalomyelitis (ADEM) in childhood, which was alleviated by DBS. Using a patient-specific connectome analysis, we sought to characterise the fibres and circuits affected by stimulation.

**Case report:**

We report a case of a 20-year-old man with progressive dystonia, myoclonic jerks, and impaired concentration following childhood ADEM. Motor assessments utilising the Unified Myoclonus Rating Scale (UMRS) and the Burke-Fahn-Marsden Dystonia Rating Scale (BFMDRS) revealed a greater improvement in dystonia compared to myoclonus following adjustments of DBS parameters. These adjustments were based on visualisation of electrode position and volume of tissue activated (VTA) 3 years after surgery. A patient-specific connectome analysis using the VTA as a region of interest revealed fibre tracts connecting to the cerebello-thalamo-cortical network and the superior frontal gyrus in addition to basal ganglia circuits as particularly effective.

**Conclusion:**

Globus pallidus internus (GPi) DBS shows promise as a treatment for secondary myoclonus-dystonia syndromes. Personalised structural considerations, tailored to individual symptoms and clinical characteristics, can provide significant benefits. Patient-specific connectome analysis, specifically, offers insights into the structures involved and may enable a favourable treatment response.

## 1. Introduction

Acute disseminated encephalomyelitis (ADEM) is a neuroinflammatory disorder characterised by central nervous system demyelination. Whereas the exact pathomechanisms remain elusive, the thalamus and the basal ganglia are commonly affected (1). Involvement of pathways within these motor circuits may result in complex secondary movement disorders (2), which necessitate tailored treatments according to individual clinical characteristics. Meanwhile deep brain stimulation (DBS) targeting these pathological network dynamics may also provide valuable insights into symptom-specific structural correlates, given its efficacy to a plethora of movement disorders such as dystonia (3) and hereditary myoclonus-dystonia (4). However, due to clinical heterogeneity and limited systematic analyses, accurately predicting outcomes of DBS for secondary dystonia syndromes is challenging. This uncertainty in prediction may lead to the cautious application of DBS beyond routine clinical practise, potentially resulting in the withholding of this effective treatment from certain patients (5). This report presents a case of secondary myoclonus-dystonia syndrome following ADEM in childhood, alleviated by DBS surgery at the internal part of the globus pallidus (GPi). We used a patient-specific connectome analysis to investigate the fibres and circuits modulated by stimulation.

## 2. Case presentation

We evaluated a 20-year-old male patient with a history of progressive dystonia, myoclonic jerks, speech difficulties, and impaired concentration. He was diagnosed with ADEM at the age of 10 in November 2009 and underwent several weeks of intensive medical therapy followed by months of rehabilitation. While initial symptoms (meningismus, encephalopathy, and cranial neuropathy) resolved, dystonic symptoms first appeared in December 2009, manifesting as involuntary movements in his right index finger while writing. Over the course of 3 years, the patient’s motor symptoms progressed beyond dystonia to myoclonic gestures.

**Figure 1:**
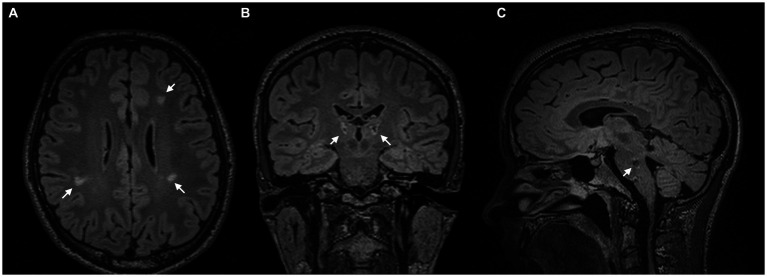
MRI scans. **(A)** Axial FLAIR-weighted MRI scans demonstrate periventricular white matter lesions around the lateral ventricles. **(B)** Coronal FLAIR-weighted MRI with bithalamic hyperintense lesions. **(C)** Sagittal FLAIR-weighted MRI reveals a hypointense lesion in the pons.

On initial examination, the patient presented with involuntary movements of the right arm and face, which were characterised by focal dysrhythmic jerks that were exacerbated by certain postures, movements, and stimuli. He also presented with multifocal dystonia of the right arm and foot. Brain MRI revealed lesions in the pons, cerebellum, thalamus, and periventricular white matter around the lateral ventricles (Figure 1).

Despite the often self-limiting nature of ADEM, we hypothesised an association between focal demyelination in the thalamus (6–8) and brainstem (7, 8) and delayed onset of symptoms (Figures 1B,C). Especially in secondary dystonia, the time between structural lesions and the onset of symptoms can be several months or even years (7). In addition, there was no family history of neurological disorders in the entire family.

The patient was thus diagnosed with a secondary multifocal myoclonus-dystonia syndrome following ADEM. Dystonia and myoclonus had remained refractory to anticonvulsants (Piracetam, Levetiracetam) and anticholinergics (Trihexyphenidyl). Following thorough multidisciplinary evaluation, the patient decided to undergo unilateral lead placement in the left GPi using directional leads (Vercise Cartesia™ 2202-02 leads, Boston Scientific Neuromodulation Corporation, Valencia, United States). DBS programming according to monopolar review revealed the largest therapeutic window when the current was steered in the anterior direction of the distal three-segmented ring contact. Using a top-down approach, we gradually increased the stimulation amplitudes to the maximum tolerated voltage while maintaining a frequency of 130 Hz and a pulse width of 60 μs. The patient was allowed to adapt DBS settings according to his preferences and was informed about the known long-term effects in dystonia. He deliberately chose programme 2 [C+ 1−(30%), 2−(35%), 3−(35%), 4 mA, 60 μs, 130 Hz] because it provided the most significant relief of motor symptoms (Table 1).

**Table 1:** DBS programming parameters.

	GPi DBS				
	Left				
Date	Programme	Active contacts	Amplitude (mA)	Pulse width (μs)	Frequency (Hz)
July 2019	1	C+ 1−(33%), 2−(33%), 3−(33%)	0.9	60	130
October 2019	2	C+ 1−(30%), 2−(35%), 3−(35%)	4	60	130
	3	C+ 2−(50%), 3−(50%)	1.8	90	130
	4	C+ 2−(50%), 3−(50%)	2	60	185
February 2023	5	C+ 2−(10%), 4−(10%), 5−(40%), 7−(40%)	2	90	185
	6	C+ 1−(30%), 2−(35%), 3−(35%)	3.5	90	130
	7	C+ 1−(30%), 2−(35%), 3−(35%)	5	60	185
	8	C+ 1−(30%), 2−(35%), 3−(35%)	3	90	185

During 3 years of follow-up, the patient reported worsening of dystonia and myoclonic jerks. To address these issues, we introduced four new stimulation programmes. In view of reports indicating efficacy of higher stimulation frequencies and pulse widths in the treatment of dystonia (9), increased pulse width (90 μs) was used in programme 6, higher frequency (185 Hz) in programme 7, and a combination of increased pulse width and frequency in programme 8, allowing the patient to adjust the settings prospectively. In addition, we used an imaging-guided optimisation of DBS parameters (programme 5) by directing the volume of tissue activated (VTA) towards proximal contacts moving from [C+, 1−(30%), 2−(35%), 3−(35%)] to [C+, 2−(10%), 4−(10%), 5−(40%), 7−(40%); avoiding contacts 3 and 6, respectively] in order to maximise the intersection between VTA and the ventral parts of the GPi, which are in close anatomical proximity to the pallidothalamic tract (PTT) (10). For dystonia, modulation of the PTT has been demonstrated to improve symptoms after pallidal DBS (11), while relief of myoclonus has been reported after contralateral pallidothalamic tractotomy (12). For this programme, we set the pulse width to 90 μs and the frequency to 185 Hz. A figure illustrating the changes in active DBS contacts after imaging-guided programming is provided as Supplementary material.

**Figure 2:**
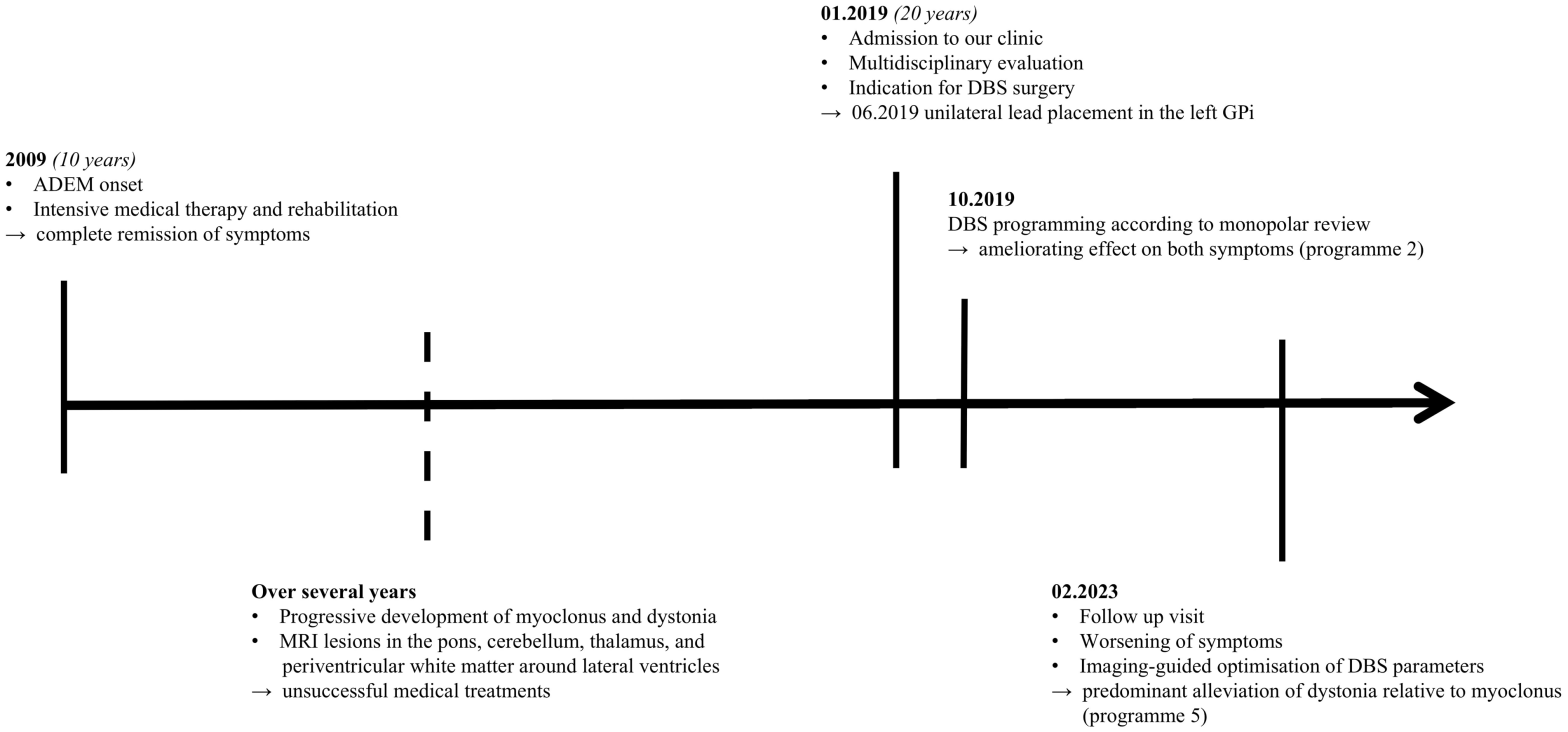
Timeline of medical history. ADEM, Acute disseminated encephalomyelitis; MRI, Magnetic resonance imaging; DBS, Deep brain stimulation; GPi, Globus pallidus internus.

We assessed motor symptoms employing the Burke-Fahn-Marsden Dystonia Rating Scale (BFMDRS) and the Unified Myoclonus Rating Scale (UMRS) with DBS turned off and on. This resulted in predominant improvement in dystonia (OFF: 26, ON: 4) relative to myoclonus (OFF: 85, ON: 60) after imaging-guided adjustments of DBS parameters. See Figure 2 for a timeline of the patient’s medical history.

### 2.1. Patient-specific connectome analysis

Magnetic resonance imaging data were acquired 1 day before surgery on a 3 T scanner. The preoperative imaging protocol included b-values of 0 and 1,000 s/mm^2^ and 30 independent diffusion gradient directions. The DBS lead and VTA visualisation was carried out using the LEAD-DBS software^1^ (13, 14). Specifically, we co-registered preoperative diffusion tensor imaging (DTI) to T1 imaging with SPM12^2^ and subsequently used Advanced Normalisation Tools (ANTs) to co-register the results with the postoperative CT scan (15). The Precise and Convenient Electrode Reconstruction for Deep Brain Stimulation (PaCER algorithm) (16) facilitated the electrode detection including its rotation. Structural connectivity was analysed using the Generalised Q-Sampling method implemented in DSI Studio^3^ (17). The VTA served as a region of interest (ROI) for identifying connected fibre tracts, with an electric field threshold of 0.2 V/mm applied during estimation (18, 19). Using a finite element model, the stimulation volume was computed by estimating the gradient distribution of the electrical charge in space on a tetrahedral mesh that differentiated four compartments: grey and white matter, electrode contact, and insulation (20), as implemented in LEAD-DBS.

**Figure 3:**
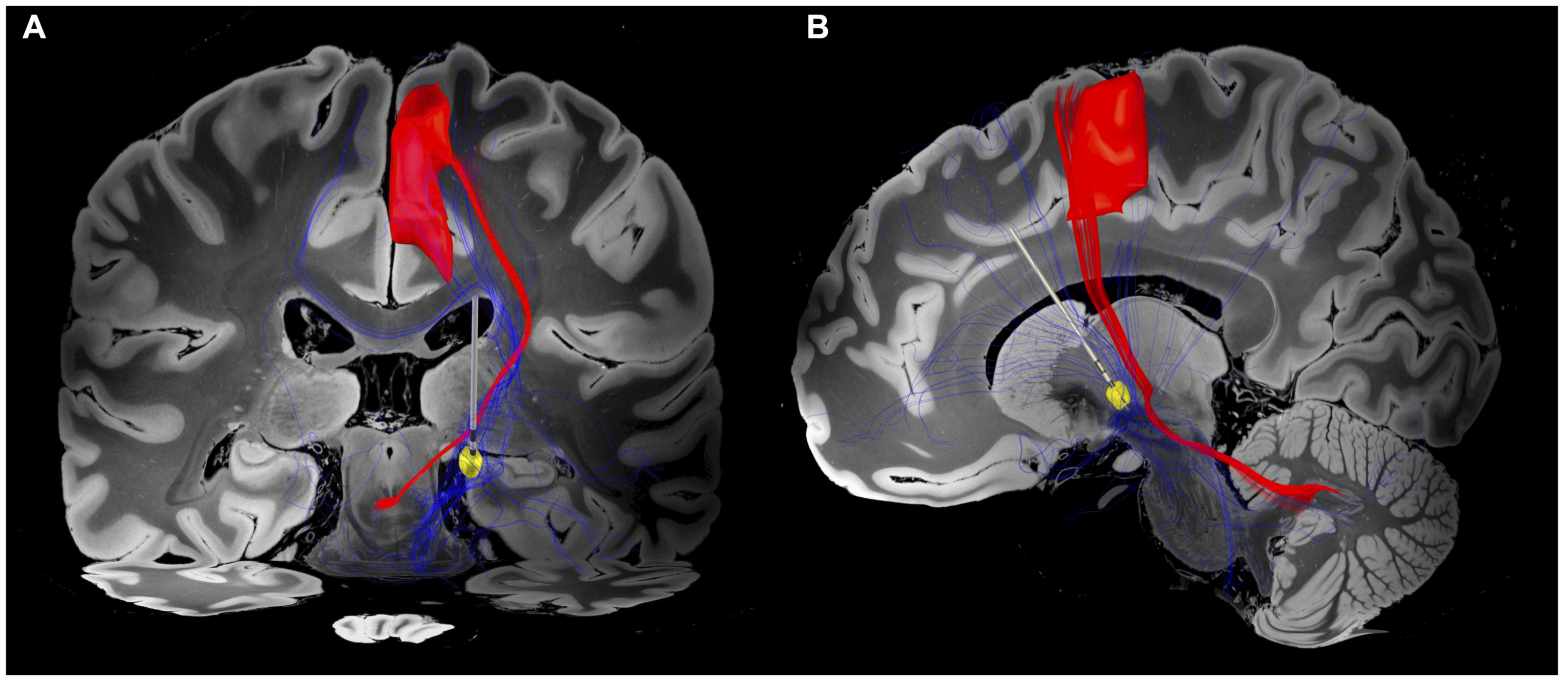
Electrode position and VTA associated fibre tracts. **(A)** Coronal view. Patient-specific connectome with VTA (yellow circle) associated fibre tracts (blue tractography) overlapping the dentato-rubro-thalamic tract (DRT; red tractography) and projecting to the superior frontal gyrus (SFG). **(B)** Sagittal view. Fibre tracts extend to prefrontal and sensorimotor regions. The closest spatial proximity to the DRT appears to be within cerebello-pallidal and pallido-thalamic connections. The VTA was generated based on the DBS parameters of programme 5 (Table 1). The DBS tractography atlas (23) and the Brainnetome atlas (22) were utilised to define DRT and SFG, respectively.

Utilising the DISTAL Minimal Atlas to define parcellation of grey matter structures (21), the VTA mostly overlapped with the prefrontal and sensorimotor subregions of the GPi (Supplementary Figure 2). As expected, our findings showed that the fibre tracts associated with the VTA of GPi DBS were connected to basal ganglia circuits and the superior frontal gyrus (SFG), which was characterised by the A6m region of the Brainnetome atlas (22). Furthermore, these fibre tracts were found to overlap with the dentato-rubro-thalamic tract (DRT), potentially indicating a modulation of the cerebello-thalamo-cortical network, as identified by the DBS tractography atlas (23) (Figure 3).

## 3. Discussion

To the best of our knowledge, this is the first published case of secondary myoclonus-dystonia syndrome after ADEM successfully treated with unilateral GPi DBS. Adjustments of DBS parameters according to connectomic data for a modulation of distributed brain networks (24) has been used to identify stimulation sweet spots in patients suffering from movement disorders such as Parkinson’s disease (25, 26) and paediatric dystonia (27). In our patient, predominant improvement of dystonia relative to myoclonus was observed when the VTA was steered to overlap prefrontal and sensorimotor subregions of the GPi. This is consistent with recent findings suggesting that pallidal DBS effectively normalises dystonia-associated sensorimotor and prefrontal hyperactivity, which have been implicated in the pathophysiology of dystonia (28).

For secondary movement disorders, growing evidence highlights a key role of alterations in multiple anatomical pathways (29). For dystonia and myoclonus in particular, desynchronisation of the cortico-basal ganglia-cerebellar network has been put forward as an underlying mechanism (30). Our results indicate an alleviation of both symptoms upon modulation of basal ganglia circuits with fibre tracts intersecting the DRT and projecting to the SFG. The interpretation of a complex multinetwork model seems to be corroborated by findings indicating a synchronicity of pallidal oscillations and myoclonic jerks (31), whereas secondary myoclonus may also result from lesions in the cerebellum (32).

While DBS targeting the posterior part of the ventrolateral thalamic nucleus (VLp) can effectively address myoclonus (33, 34), in our patient, right-sided dystonic features were the predominant symptom, causing significant difficulties with activities of daily-living and severely affecting the patient’s quality of life. This led us to choose the left GPi as the most appropriate target for DBS treatment, given its widely demonstrated efficacy for ameliorating dystonia (3). While this case highlights the importance of a thorough clinical assessment as the basis for selecting stimulation targets, future insights from patient-specific connectivity profiles could improve our understanding of the underlying neural dynamics and provide insights into the precise target for DBS leads in rare movement disorders.

### 3.1. Limitations

While this case report demonstrates the long-term effects of pallidal DBS in a secondary myoclonus-dystonia syndrome and thereby highlights the underlying networks, there are some limitations. Although imaging-guided optimisation of DBS parameters was carried out based on the VTA and its adjacent neuroanatomical structures, the stimulation parameters were not refined using the tractography results from the patient-specific connectome analysis. In addition, our preoperative imaging protocol prevented us from testing for abnormal kurtosis measures. Diffusion kurtosis imaging (DKI) has been shown to be more sensitive to microstructural changes in demyelinating disorders (35) by quantifying the deviation of the water diffusion displacement profile from the Gaussian distribution (36). Another limitation is reflected in the absence of functional data, which may have provided supplementary insights into alterations in neural connectivity. Finally, this report describes a single case and therefore the findings may not be generalisable to a broader population. Despite these limitations, future studies could explore the potential of using patient-specific connectome analyses to fine-tune DBS parameters for movement disorders, tailoring the treatment based on individual symptoms and clinical characteristics.

## Data availability statement

The datasets presented in this article are not readily available because of ethical and privacy restrictions. Requests to access the datasets should be directed to the corresponding author.

## Ethics statement

Ethical review and approval was not required for this case report in accordance with the local legislation and institution requirements. The patient provided written informed consent for the use of anonymized data for research purposes and for publication of any potentially identifiable images or data included in this article.

## Author contributions

AC and LB collected the clinical data and co-wrote the manuscript. AC performed the image analysis. MG, MB, and CN contributed to the data curation. LT and DP confirmed the diagnosis of the case and supervised the study. All authors contributed to the article and approved the submitted version.

## Footnotes

^1^ https://www.lead-dbs.org

^2^ http://www.fil.ion.ucl.ac.uk/spm/software/

^3^ https://dsi-studio.labsolver.org
