# Supplementary material for: Case report: Unilateral GPi DBS in secondary myoclonus-dystonia syndrome after acute disseminated encephalomyelitis
Source: Front Neurol. 2023 Sep 26;14:1238743. doi: 10.3389/fneur.2023.1238743 (PMC10562570; doi:10.3389/fneur.2023.1238743)
Supplement: Supplementary file 1 [file Table_1.DOCX]

Supplementary Material


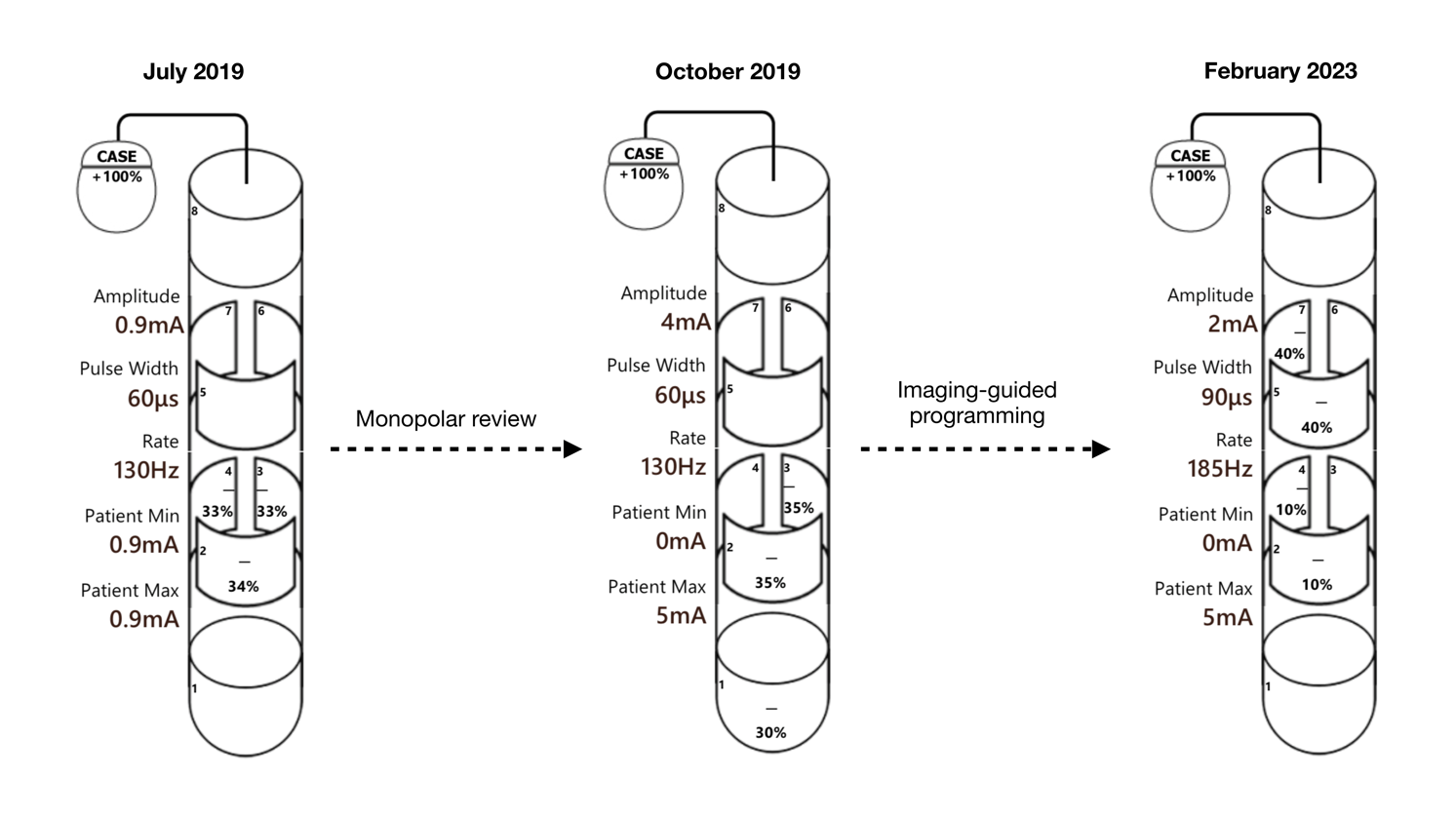
**Supplementary Figure 1: Chronology of DBS parameter adjustments**

**
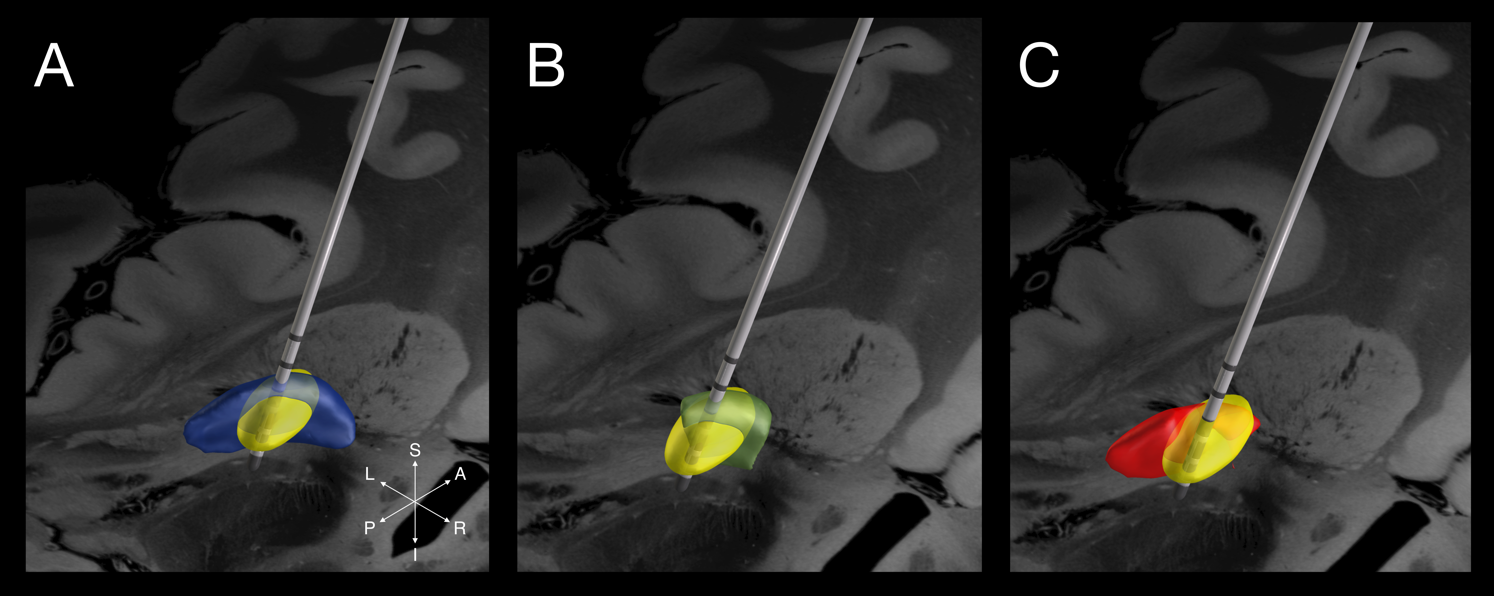
Supplementary Figure 2: Overlap of the VTA with the GPi and its subregions.** A) VTA (yellow) overlapping the GPi (blue). B) Prefrontal subregion overlap (green). C) Sensorimotor subregion overlap (red). Parcellation of the GPi was defined utilising the DISTAL Minimal Atlas.
